# Supplementary material for: Single-residue mutation in protein kinase C toggles between cancer and neurodegeneration
Source: Biochem J. 2023 Aug 25;480(16):1299–316. doi: 10.1042/BCJ20220397 (PMC10586763; doi:10.1042/BCJ20220397)

Supplementary Figure 1

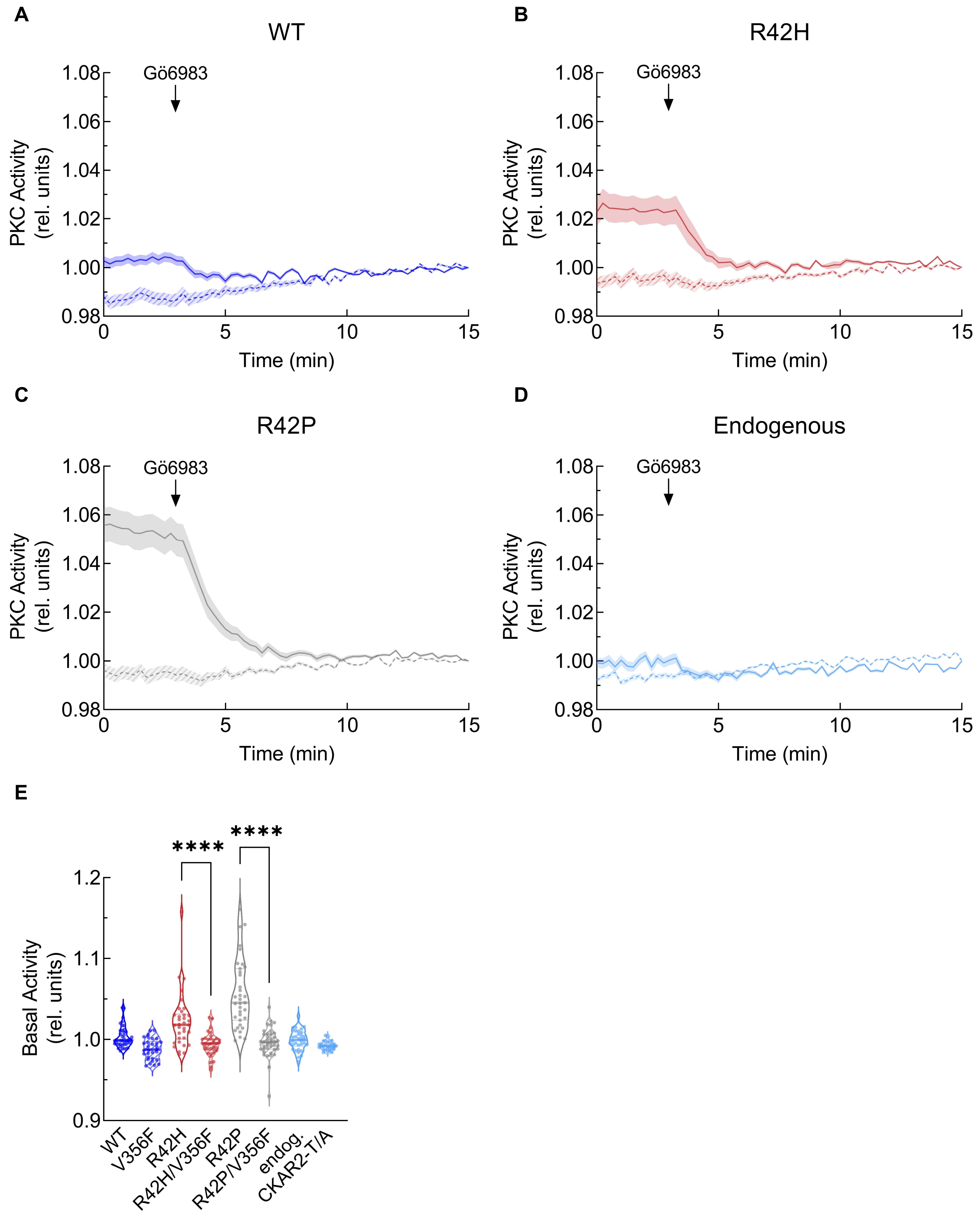

Supplementary Figure 2

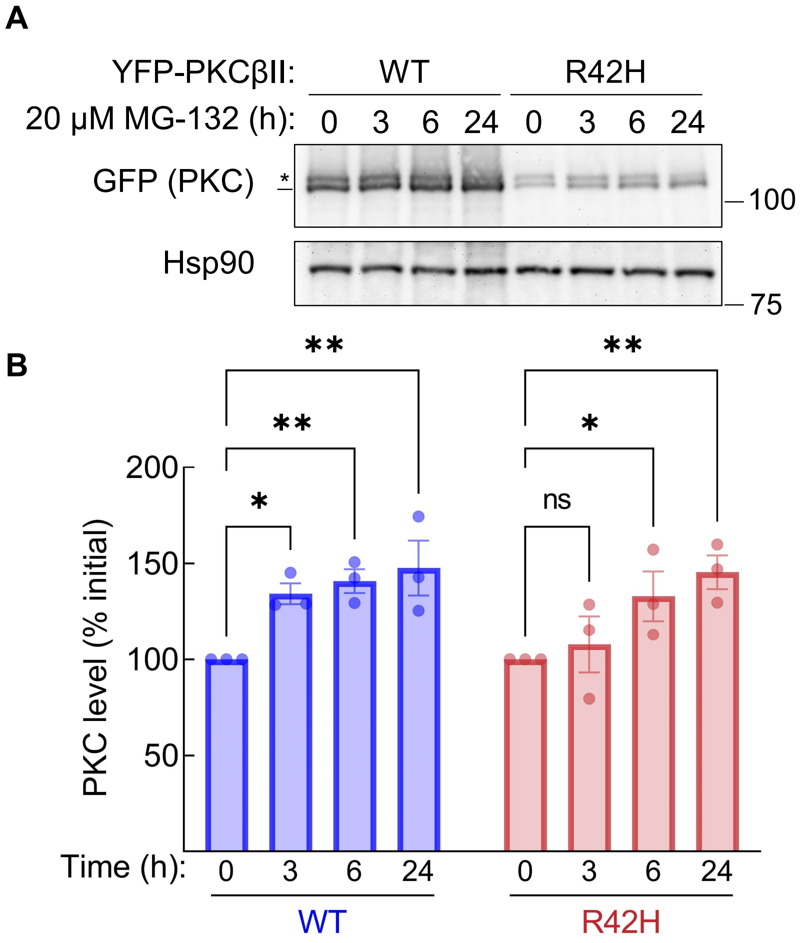

Supplementary Figure 3

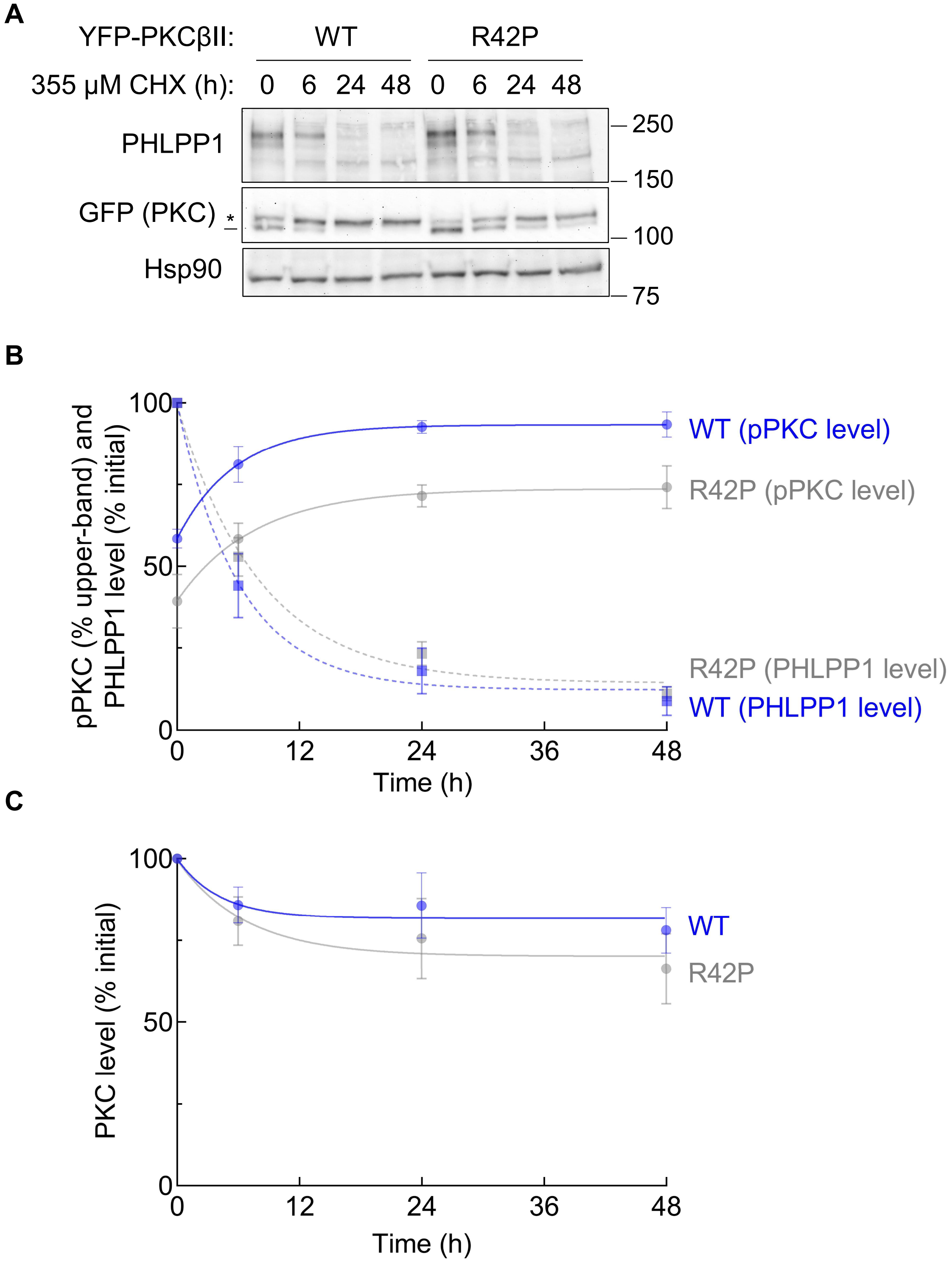

Supplementary Figure 4

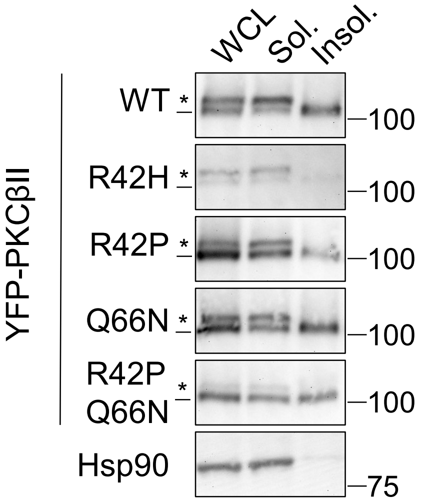

Supplementary Figure 5

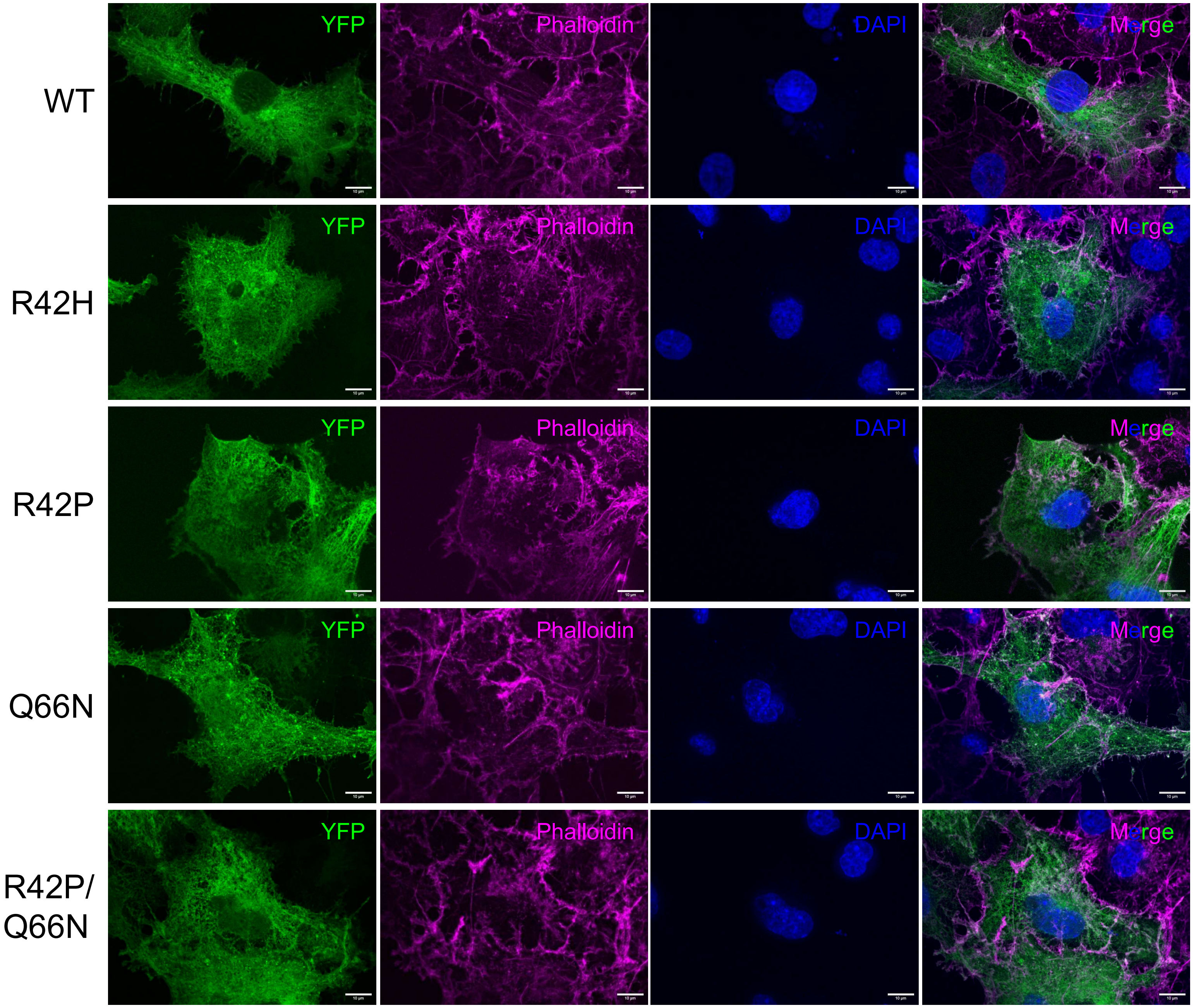

Supplement: Supplementary Material [file BCJ-480-1299-s1.pdf]
